# Casein improves brachial and central aortic diastolic blood pressure in overweight adolescents: a randomised, controlled trial

**DOI:** 10.1017/jns.2013.29

**Published:** 2014-01-02

**Authors:** Karina Arnberg, Anni Larnkjær, Kim F. Michaelsen, Signe Marie Jensen, Camilla Hoppe, Christian Mølgaard

**Affiliations:** 1 Department of Nutrition, Exercise and Sports, University of Copenhagen, Rolighedsvej 30, 1958 Frederiksberg, Denmark; 2 The National Food Institute, Technical University of Denmark, Mørkhøj Bygade 19, 2860 Søborg, Denmark

**Keywords:** Whey, Casein, Overweight children: Blood pressure, ACE, angiotensin-I-converting enzyme, Aix, augmentation index, BP, blood pressure, CRP, C-reactive protein, DBP, diastolic blood pressure, PWV, pulse wave velocity, SBP, systolic blood pressure

## Abstract

Arterial stiffness, blood pressure (BP) and blood lipids may be improved by milk in adults and the effects may be mediated via proteins. However, limited is known about the effects of milk proteins on central aortic BP and no studies have examined the effects in children. Therefore, the present trial examined the effect of milk and milk proteins on brachial and central aortic BP, blood lipids, inflammation and arterial stiffness in overweight adolescents. A randomised controlled trial was conducted in 193 overweight adolescents aged 12–15 years. They were randomly assigned to drink 1 litre of water, skimmed milk, whey or casein for 12 weeks. The milk-based test drinks contained 35 g protein/l. The effects were compared with the water group and a pretest control group consisting of thirty-two of the adolescents followed 12 weeks before the start of the intervention. Outcomes were brachial and central aortic BP, pulse wave velocity and augmentation index, serum C-reactive protein and blood lipids. Brachial and central aortic diastolic BP (DBP) decreased by 2·7% (*P* = 0·036) and 2·6 % (*P* = 0·048), respectively, within the casein group and the changes were significantly different from those of the pretest control group (*P* = 0·040 and *P* = 0·034, respectively). There was a significant increase in central aortic DBP, and in brachial and central systolic BP in the whey group compared with the water group (*P* = 0·003, *P* = 0·009 and *P* = 0·002, respectively). There were no changes in measures of arterial stiffness or blood lipid concentrations. A high intake of casein improves DBP in overweight adolescents. Thus, casein may be beneficial for younger overweight subjects in terms of reducing the long-term risk of CVD. In contrast, whey protein seems to increase BP compared with drinking water; however, water may be considered an active control group.

The prevalence of overweight has increased in the past decades among children in the Western world^(^^1^^,^^2^^)^. Overweight children have higher blood pressure (BP) and abnormal blood lipid levels compared with normal-weight children^(^^3^^,^^4^^)^, and the risk factors track from childhood into adulthood^(^^5^^)^. *Post mortem* studies have found atherosclerotic lesions in children and the extent of the lesions has been related to the number of cardiovascular risk factors including high BMI, raised systolic BP (SBP) and diastolic BP (DBP) and abnormal blood lipid concentrations^(^^6^^)^. Also, overweight children have been shown to have increased arterial stiffness and endothelial dysfunction compared with normal-weight children^(^^7^^,^^8^^)^.

Milk is an important source of protein in the Western diet and epidemiological studies have shown inverse associations between dairy consumption and metabolic syndrome risk factors in children and adults^(^^9^^–^^11^^)^. Also, intervention studies in overweight or hypertensive adults have shown improvements in measures of arterial stiffness and brachial BP by milk proteins^(^^12^^–^^15^^)^, and a meta-analysis of randomised controlled trials concluded that milk-derived tripeptides have a hypotensive effect in hypertensive adults^(^^16^^)^. Central aortic BP is a better predictor of cardiovascular events than brachial BP^(^^17^^)^ and a recent study in hypertensive adults showed improvements in central aortic BP following casein tablets^(^^18^^)^. The mechanisms whereby milk and milk proteins may affect BP and arterial stiffness have been linked to the angiotensin-I-converting enzyme (ACE). Thus, *in vitro* studies have found ACE-inhibitory peptides in the amino acid sequences of whey and casein^(^^19^^)^.

The blood lipid profile has been improved by longer-term intake of whey protein in overweight adults^(^^20^^)^. The mechanism has been related to the leucine content, which in an animal study has been found to decrease hepatic cholesterol synthesis and thereby decrease total plasma cholesterol and LDL-cholesterol^(^^21^^)^. Also *in vitro*, milk proteins have been shown to down-regulate genes involved in intestinal fatty acid and cholesterol synthesis and transport^(^^22^^)^.

Since the early abnormalities preceding atherosclerosis occur in childhood, we find it highly relevant to study the potential beneficial effects of supplementing with dairy products in young people. Therefore, in the present study we examined whether there are beneficial effects on brachial and central aortic BP, blood lipids, inflammation and arterial stiffness in overweight adolescents with a low habitual milk intake by increasing the intake of low-fat milk and we examined whether potential effects are mediated by whey or casein.

## Method

### Subjects

Overweight and obese adolescents aged 12–15 years with an age- and sex-adjusted BMI corresponding to adult BMI > 25 kg/m^2(^^23^^)^ were recruited from November 2008 to December 2010 through mailed invitations. Invitations were sent to all children of birth years 1995, 1996, 1997 and 1998 living in the Copenhagen area using extractions from the National Danish Civil Registration. The exclusion criteria were milk and yogurt intake >250 ml/d, smoking, chronic diseases and consumption of antibiotics within the last month before the start of the intervention^(^^24^^,^^25^^)^.

### Study design and methodology

The study was a randomised parallel intervention study conducted at the Department of Nutrition, Exercise and Sports, University of Copenhagen. The overall aim of the intervention was to study the effects of skimmed milk, whey and casein compared with drinking water and compared with a pretest control group on risk factors of the metabolic syndrome in overweight adolescents. In order to assess the effect of supplementing with skimmed milk, whey and casein, the study was designed to include control groups not consuming extra energy. Thus, a water group was used as a control but because water may be an active control^(^^26^^)^, the study was also designed to include a control group consuming no test drink. Therefore, a subgroup of the children was followed for 12 weeks before starting the intervention (corresponding to a pretest control group measured at time –12 weeks) and randomised to drink 1 litre/d for 12 weeks of: water, skimmed milk, whey or casein. All adolescents were examined before the start of the intervention (week 0) and after 12 weeks of intervention (week 12). All examinations and measurements were obtained in the fasting state.

The study was conducted according to the guidelines laid down in the Declaration of Helsinki and all procedures involving human subjects were approved by the Scientific Ethics Committees of the Capital Region of Denmark (journal no. H-A-2008-084). Written informed consent was obtained from all the parents and the trial was registered at ClinicalTrials.gov (NCT00785499).

### Test products

The composition of the test drinks is given in Table 1. All test drinks were ready to drink. The whey and casein drinks were based on intact protein (whey protein isolate (Lacprodan DI-9213) and calcium caseinate (Miprodan 40)). All milk-based test drinks were produced by ARLA Food Ingredients. The protein content in all milk-based test drinks was 35 g/l. The skimmed milk, whey and casein drinks were packaged in identical 200 ml milk cartons and coded by ARLA Food Ingredients. At the Department of Nutrition, Exercise and Sports, the water and milk-based test drinks were recoded using a letter for each drink by a technical assistant, who was otherwise not involved in the study. The milk-based test drinks were provided to the children at the start of the intervention and half way through the intervention and the children were told to drink five cartons during the day. The skimmed milk, whey and casein groups were blinded for participant and investigator. The water test drink was bottled water produced by Jørgensen Engros A/S. The water was packaged in 500 ml plastic bottles, and all bottles were provided to the participants at the start of the intervention and the children were told to drink two bottles during the day. The water group was not blinded because the children clearly could taste whether they drank water or a milk-based test drink. The children were told to eat *ad libitum* and maintain their usual physical activity levels during the study.

**Table 1.** Average nutritional composition of the test drinks

Nutritional content	Water	Skimmed milk	Casein	Whey
Energy (kJ/100 g)	–	156	136	137
Fat (g/100 g)	–	0·47	0·05	0·04
Lactose (g/100 g)	–	4·68	4·44	4·45
Protein (g/100 g)	–	3·47	3·46	3·48
Casein:whey ratio		80:20	100:0	0:100
Amino acids (mol%)	–			
Asp	–	7·76	6·83	10·96
Thr	–	4·53	4·13	7·75
Ser	–	7·11	7·22	6·12
Glu	–	19·07	19·55	16·12
Pro	–	10·73	12·02	6·6
Gly	–	3·19	3·06	2·74
Ala	–	4·77	4·26	7·06
Tp Cys	–	0·86	0·31	2·68
Val	–	6·46	6·80	6·23
Met	–	2·27	2·39	1·88
Ile	–	4·77	4·71	6·23
Leu	–	9·47	9·09	10·24
Tyr	–	3·40	3·90	2·00
Phe	–	3·57	3·86	2·26
His	–	2·26	2·35	1·39
Lys	–	7·34	6·91	8·41
Arg	–	2·46	2·61	1·35
Na (mg/100 g)	0·8	30·0	140·0	10·0
P (mg/100 g)	–	100·0	40·0	60·0
Ca (mg/100 g)	3·1	120·0	60·0	10·0
Mg (mg/100 g)	0·2	10·0	0·0	0·0
K (mg/100 g)	0·1	160·0	90·0	10·0

–, Data were not obtained.

### Compliance

The adolescents were told to record their consumption of test drinks in booklets with calendar tick boxes and to count the number of leftover water bottles or milk cartons. Moreover, serum urea-N was analysed as a measure of recent protein intake^(^^27^^)^ using the kinetic UV assay on Pentra 400 analysers (Horiba ABX) with intra- and inter-assay variations of 1·0 and 5·3 %, respectively.

### Pubertal development

Tanner stage was assessed at the start of the intervention using self-administrated questionnaires^(^^28^^,^^29^^)^.

### Anthropometry

Examinations were conducted in the fasting state. Weight was recorded on a digital scale to 0·1 kg accuracy (Tanita BWB600; Tanita) in underwear and a cotton T-shirt after the bladder had been emptied. Height was measured to the nearest 0·01 cm without shoes using a wall-mounted digital stadiometer in triplicate (235 Heightronic Digital Stadiometer; Quick Medical and Measurement Concepts).

### Measurement of plasma lipids and C-reactive protein

As also described previously^(^^24^^)^, serum TAG, serum total cholesterol, serum HDL-cholesterol and serum LDL-cholesterol were analysed using the specific ABX Pentra kits on Pentra 400 analysers (Horiba ABX). The intra-assay and inter-assay variations of the analysis of serum TAG were 2·6 and 3·2 %, of total cholesterol 0·9 and 1·6 %, of HDL-cholesterol 1·2 and 4·0 %, and of LDL-cholesterol 1·3 and 2·7 %, respectively. Serum C-reactive protein (CRP) concentrations were analysed using the specific high-sensitivity Horiba ABX CRP CP Assay on Pentra 400 analysers with a detection limit of 0·10 mg/l. The intra- and inter-assay variations were 3·6 and 8·1 %, respectively. For serum CRP, data below the detection limit of 0·10 were set at 0·05 (ten at week –12, forty-four at week 0 and thirty-two at week 12).

### Haemodynamics

All haemodynamic measures were obtained after a 10-min rest in the supine position. Brachial BP was measured three times using an automatic digital BP device (model UA-787 SN 50802 00005, Kivex; A&D Medical). The average of the last two measures was used. The CV were 5·4 and 10·7 % for SBP and DBP, respectively. Pulse wave analysis was used to derive measures of the augmentation index (Aix), central aortic SBP and DBP. Radial pulse waves were obtained by placing an applanation tonometer over the right radial using the SphygmoCor System (Atcor Medical). This method of obtaining central aortic pressures has been validated against invasive methods^(^^30^^)^. The CV of central SBP and central DBP were 1·5 and 0·8 %, respectively. Aix is an index of the enhancement of the aortic systolic pressure generated by the return of the reflected waves^(^^31^^)^. Aix is affected by heart rate^(^^32^^)^ and the values used were those adjusted by the software to a standard of 75 beats per min. The coefficient of repeatability of Aix was 12 %. Pulse wave velocity (PWV) was recorded at the carotid and femoral sites and calculated by the software as: PWV = D/Δt (m/s). D was the difference in distance between the carotid and femoral sites measured using a measuring tape at the surface distances: (1) the suprasternal notch and the femoral pulse; and (2) the suprasternal notch and the carotid pulse. Δt was the time difference between the two measuring sites calculated at the foot of the measured pulse wave in relation to the foot of the electrocardiogram (ECG) waveform. The coefficient of repeatability of PWV was 0·82 m/s and the CV of PWV was 8·5 %.

### Statistical analysis

Values presented are means and standard deviations for normally distributed variables and medians and interquartile ranges for variables that are not normally distributed. Differences in characteristics between the four test-drink groups at the start of the intervention (week 0) were tested by the χ^2^ test, one-way ANOVA or the Kruskall–Wallis test. Baseline *post hoc* pairwise comparisons were conducted with the significance level corrected for multiple testing. Outcome variables were brachial and central aortic SBP and DBP, PWV and Aix, serum TAG, serum cholesterol, serum LDL-cholesterol, serum HDL-cholesterol and serum CRP. Serum cholesterol, LDL-cholesterol, TAG and CRP were log-transformed before all analyses. Changes within the 12-week periods were compared by paired *t* tests and for serum CRP changes were compared by the Wilcoxon sign-rank test. Correlations between brachial BP and central aortic BP were calculated using the Pearson correlation test. Compliance was calculated as the percentage of planned intake. Intake estimated from the number of leftovers was used when the booklet was missing. To assess the effect of skimmed milk, whey and casein compared with water and compared with the pretest control group, a mixed linear model was fitted by xtmixed in STATA (StataCorp LP). The model has also been described elsewhere^(^^25^^)^. The following predictors were included in the model: time (week –12, week 0, week 12), intervention time (week 0, week 12) and the intervention group × intervention time interaction. Thus, for all participants a general time effect (called ‘time’) was assumed corresponding to the underlying change happening over time with no intervention, i.e. in the pretest control group. In addition, an intervention time effect (called ‘intervention time’) was included for each test-drink group (intervention time × group interaction) during the intervention period only, corresponding to the additional change happening over time due to the intervention. For a given intervention group the effect of intervention time corresponded to the effect compared with the pretest control. The interaction term was used to assess the effect of each test drink compared with that in the water group using the latter as the reference group in the intervention group variable. We have previously shown that milk, whey and casein increase age-adjusted BMI^(^^25^^)^. Therefore, for each outcome, a second model was constructed which also included: BMI, sex, Tanner stage and age. Sex × test-drink interactions were tested and, if non-significant, the interaction term was removed from the model. Since the repeated measurement model is based on available case analysis, data on all subjects measured at the different time points are shown in the tables. *P* values below 0·05 were considered significant. All analyses were performed using STATA 11.0 (StataCorp LP).

### Study size and power calculation

The primary outcomes of the study were body composition and fasting insulin, glucose, blood lipids and CRP whereas the haemodynamic measures were secondary outcomes. Body weight was used to power the study. A sample size of 200 children with an expected drop-out of 10 % was selected in order to observe a difference of 0·4 sd, which was assumed to correspond to a weight difference of 1 kg with a significance level of <0·05 and a power of 80 % for each treatment comparison. The power calculation was performed using the method of Altman^(^^33^^)^ and the method implies that the study is powered to detect a pairwise difference of 0·4 sd in any of the outcomes, should it exist.

## Results

### Subjects

A total of 203 adolescents commenced the trial but ten withdrew before the start of the intervention. Thus, 193 children (62 % girls) were examined at week 0 (fifty water, forty-seven casein, forty-eight skimmed milk, forty-eight whey), and, of those, thirty-two subjects participated in the pretest control group. A total of twenty children withdrew during the intervention (eleven from the casein group, four from the skimmed milk group and five from the whey group); hence 173 adolescents completed the trial (fifty water, thirty-six casein, forty-four skimmed milk and forty-three whey). Table 2 shows the characteristics of the subjects at the start of the intervention. At the start of the intervention, there were no differences in baseline characteristics or in any of the primary outcomes between the four test-drink groups.

**Table 2.** Characteristics of the test-drink groups at week 0* and the pretest control group at week –12 (Mean values and standard deviations, medians and interquartile ranges (IQR) or number of subjects)

	Pretest control (*n* 32)		Water (*n* 50)		Casein (*n* 47)		Skimmed milk (*n* 48)		Whey (*n* 48)	
	Mean	sd	Mean	sd	Mean	sd	Mean	sd	Mean	sd
Sex (*n*)										
Male	12		18		18		17		20	
Female	20		32		29		31		28	
Age (years)										
Median	13·7		13·0		13·0		13·1		12·9	
IQR	13·2–14·1		12·8–13·5		12·8–13·6		12·5–13·8		12·6–13·4	
Tanner stage (*n*)										
1	0		3		2		2		0	
2	2		7		10		9		12	
3	9		20		22		17		16	
4	13		16		8		18		17	
5	8		4		5		2		3	
Weight (kg)	69·8	7·8	66·9	8·5	66·0	9·0	66·0	10·1	66·2	10·7
Height (cm)	165·5	8·3	162·9	7·6	162·3	7·7	162·4	7·5	162·8	8·7
BMI (kg/m^2^)										
Median	25·1		25·2		24·4		24·7		24·7	
IQR	24·3–26·1		23·1–27·0		23·5–26·5		23·5–25·9		23·1–26·2	
Brachial SBP (mmHg)	111·4	8·6	111·8	7·9	109·3	7·6	109·1	6·5	111·3	7·4
Brachial DBP (mmHg)	64·3	5·9	65·3	7·6	66·1	5·9	65·3	6·1	64·5	5·7
Central SBP (mmHg)†	94·2	5·8	94·8	5·3	93·6	6·0	93·6	5·7	94·5	5·4
Central DBP (mmHg)†	65·8	5·6	66·5	7·3	67·7	5·8	66·6	6·5	66·2	5·5

SBP, systolic blood pressure; DBP, diastolic blood pressure.

* There were no differences between the four test-drink groups at baseline (week 0).

† Two missing in the pretest control, water and skimmed milk groups, respectively. Three missing in the casein and whey groups, respectively.

### Compliance

Mean intake expressed in percentage of planned intake was 95, 92, 91 and 87 % for water, skimmed milk, casein and whey, respectively. Data with changes in serum urea-N concentrations have been published elsewhere^(^^25^^)^. Briefly, serum urea-N increased in the casein and skimmed milk groups compared with the pretest control and serum urea-N increased in all milk-based test-drink groups compared with the water group.

### Blood lipids

It was not possible to obtain blood samples in two adolescents from the water and skimmed milk groups, respectively, at week 12. There were no significant changes in blood lipids in any of the test-drink groups over the intervention (Table 3).

**Table 3.** BMI and concentrations of blood lipids in the pretest control group and in those drinking water, casein, skimmed milk or the whey drink* (Medians and interquartile ranges (IQR) or mean values and standard deviations)

	Week –12		Week 0		Week 12		Change†				
	Median	iqr	Median	iqr	Median	iqr	Median	iqr	Paired test‡	Treatment§	Treatment‖
BMI (kg/m^2^)¶											
Pretest control	25·1	24·3–26·1	25·3	24·6–26·6	–	–	0·3	–0·05 to 0·58	*P* = 0·042	–	–
Water	–	–	25·2	23·1–27·0	25·4	23·7–26·9	0·4	–0·24 to 0·75	*P* = 0·010	*P* = 0·321	–
Casein	–	–	24·4	23·5–26·5	25·6	24·2–26·9	0·8	0·39 to 1·24	*P* < 0·001	*P* < 0·001	*P* = 0·002
Skimmed milk	–	–	24·7	23·5–25·9	25·3	24·2–27·0	0·6	0·19 to 1·04	*P* < 0·001	*P* = 0·001	*P* = 0·022
Whey	–	–	24·7	23·1–26·2	25·5	23·8–26·7	0·7	0·28 to 1·20	*P* < 0·001	*P* < 0·001	*P* = 0·009
Cholesterol (mmol/l)**											
Pretest control	3·80	3·32–4·29	3·84	3·38–4·56	–	–	0·05	–0·09 to 0·32	*P* = 0·938	–	–
Water	–	–	4·02	3·53–4·66	4·12	3·50–4·45	0·00	–0·31 to 0·32	*P* = 0·757	*P* = 0·370	–
Casein	–	–	3·85	3·61–4·77	4·44	3·79–4·71	0·13	–0·33 to 0·43	*P* = 0·187	*P* = 0·509	*P* = 0·091
Skimmed milk	–	–	4·34	3·82–4·74	4·26	3·84–4·73	–0·05	–0·26 to 0·27	*P* = 0·775	*P* = 0·531	*P* = 0·782
Whey	–	–	4·04	3·60–4·93	4·09	3·65–4·63	0·03	–0·30 to 0·27	*P* = 0·811	*P* = 0·547	*P* = 0·757
LDL-cholesterol (mmol/l)**											
Pretest control	2·29	1·85–2·76	2·15	1·88–2·79	–	–	–0·02	–0·12 to 0·12	*P* = 0·601	–	–
Water	–	–	2·35	1·89–2·90	2·41	1·97–2·89	0·08	–0·27 to 0·26	*P* = 0·946	*P* = 0·940	–
Casein	–	–	2·38	1·98–2·72	2·68	2·13–3·10	0·06	–0·13 to 0·35	*P* = 0·051	*P* = 0·087	*P* = 0·053
Skimmed milk	–	–	2·53	1·96–3·02	2·58	2·02–2·88	0·04	–0·16 to 0·24	*P* = 0·827	*P* = 0·698	*P* = 0·609
Whey	–	–	2·25	1·95–2·76	2·26	2·02–2·71	0·06	–0·24 to 0·23	*P* = 0·700	*P* = 0·919	*P* = 0·847
HDL-cholesterol (mmol/l)**											
Pretest control									*P* = 0·707	–	–
Mean	1·16		1·17		–		0·01				
sd	0·19		0·23		–		0·16				
Water									*P* = 0·742	*P* = 0·379	–
Mean	–		1·20		1·20		−0·07				
sd	–		0·21		0·19		0·15				
Casein									*P* = 0·980	*P* = 0·408	*P* = 0·998
Mean	–		1·16		1·15		0·00				
sd	–		0·27		0·26		0·13				
Skimmed milk									*P* = 0·191	*P* = 0·118	*P* = 0·415
Mean	–		1·20		1·19		−0·03				
sd	–		0·30		0·27		0·16				
Whey									*P* = 0·720	*P* = 0·412	*P* = 0·968
Mean	–		1·26		1·26		−0·01				
sd	–		0·32		0·25		0·20				
TAG (mmol/l)**											
Pretest control	0·81	0·53–1·19	0·87	0·62–1·03	–	–	–0·04	–0·22 to 016	*P* = 0·625	–	–
Water	–	–	0·87	0·65–1·22	0·86	0·65–1·04	–0·01	–0·24 to 0·13	*P* = 0·363	*P* = 0·267	–
Casein	–	–	0·73	0·59–0·96	0·87	0·61–1·15	0·07	–0·20 to 0·32	*P* = 0·226	*P* = 0·709	*P* = 0·107
Skimmed milk	–	–	0·84	0·65–1·15	0·79	0·63–1·14	0·00	–0·20 to 0·16	*P* = 0·665	*P* = 0·617	*P* = 0·503
Whey	–	–	0·82	0·69–1·15	0·77	0·64–1·03	0·00	–0·28 to 0·14	*P* = 0·823	*P* = 0·559	*P* = 0·565

–, No measurements were performed.

* There were no differences between the four test-drink groups at baseline (week 0).

† For test drinks, calculated as the change from week 0 to week 12; in the pretest control group, calculated as the change from week –12 to week 0.

‡ Level of significance between week 0 and week 12 for the test-drink groups and between week –12 and week 0 for the pretest control group.

§ Level of significance for difference over the intervention between the test-drink group and the pretest control group.

|| Level of significance for difference over the intervention between the milk-based test drink and water.

¶ For BMI, at week –12 (*n* 32); at week 0 (water (*n* 50), casein (*n* 47), skimmed milk (*n* 48), whey (*n* 48)); at week 12 (water (*n* 50), casein (*n* 36), skimmed milk (*n* 44), whey (*n* 43)).

** For blood lipids, at week –12 (*n* 32); at week 0 (water (*n* 50), casein (*n* 47), skimmed milk (*n* 48), whey (*n* 48)); at week 12 (water (*n* 49), casein (*n* 36), skimmed milk (*n* 43), whey (*n* 43)).

### Haemodynamic parameters

Due to difficulties in measuring the children, no measurements of PWV were obtained in one subject at week –12, eleven (two water, two casein, six skimmed milk, one whey) at week 0 and nineteen (six water, four casein, eight skimmed milk, one whey) at week 12. For pulse wave analysis, no measurements were obtained in two subjects at week –12, ten (two water, three casein, two skimmed milk, three whey) at week 0 and six (three water, two skimmed milk, one whey) at week 12. At the start of the intervention, brachial SBP and DBP were 110 (sd 7) and 65 (sd 6) mmHg, respectively. Central aortic SBP, DBP, PWV and Aix were 94 (sd 6) mmHg, 67 (sd 6) mmHg, 4·78 (sd 0·72) m/s and –0·77 (sd 9·44) %, respectively. There were good correlations between brachial and central aortic DBP (*r* = 0·97; *P* < 0·001) and between brachial and central aortic SBP (*r* 0·84; *P* < 0·001).

At week 0, twenty subjects had brachial SBP ≥ 120 mmHg, four subjects had brachial DBP ≥ 80 mmHg and one subject had brachial DBP/SBP ≥ 120/80 mmHg.

Brachial DBP decreased in the casein group from week 0 to week 12 by 2·7 % (*P* = 0·036) (Table 4) and it decreased compared with the pretest control (*P* = 0·040). The effect of casein on brachial DBP compared with the pretest control remained significant after adjusting for BMI, Tanner stage, sex and age (*P* = 0·026). There were no significant effects of water, skimmed milk or whey on brachial DBP.

**Table 4.** Blood pressure in the pretest control group and in those drinking water, casein, skimmed milk or the whey drink* (Mean values and standard deviations)

	Week –12		Week 0		Week 12		Change†				
	Mean	sd	Mean	sd	Mean	sd	Mean	sd	Paired test‡	Treatment§	Treatment‖
Brachial SBP (mmHg)¶											
Pretest control	111·4	8·6	110·9	8·5	–	–	–0·5	5·8	*P* = 0·651	–	–
Water	–	–	111·8	7·9	110·8	6·6	–1·1	6·3	*P* = 0·242	*P* = 0·513	–
Casein	–	–	109·3	7·6	110·0	5·8	–0·4	5·4	*P* = 0·644	*P* = 0·729	*P* = 0·763
Skimmed milk	–	–	109·1	6·5	109·8	7·4	0·7	6·1	*P* = 0·458	*P* = 0·962	*P* = 0·431
Whey	–	–	111·3	7·4	113·7	9·7	2·0	7·8	*P* = 0·105	*P* = 0·098	*P* = 0·009
Brachial DBP (mmHg)¶											
Pretest control	64·3	5·9	63·7	6·1	–	–	–0·6	4·3	*P* = 0·443	–	–
Water	–	–	65·3	7·6	63·8	5·8	–1·5	5·6	*P* = 0·067	*P* = 0·056	–
Casein	–	–	66·1	5·9	63·9	5·7	–1·8	5·0	*P* = 0·036	*P* = 0·040	*P* = 0·767
Skimmed milk	–	–	65·3	6·1	64·7	5·6	–0·5	5·7	*P* = 0·558	*P* = 0·294	*P* = 0·342
Whey	–	–	64·5	5·7	65·2	5·6	0·6	5·9	*P* = 0·498	*P* = 0·793	*P* = 0·061
											
Central SBP (mmHg)**											
Pretest control	94·2	5·8	93·7	6·5	–	–	–0·5	4·9	*P* = 0·604	–	–
Water	–	–	94·8	5·3	93·8	4·8	–1·0	4·4	*P* = 0·117	*P* = 0·291	–
Casein	–	–	93·6	6·0	93·9	5·4	0·1	5·1	*P* = 0·935	*P* = 0·766	*P* = 0·440
Skimmed milk	–	–	93·6	5·7	92·8	5·9	–0·7	4·5	*P* = 0·348	*P* = 0·274	*P* = 0·930
Whey	–	–	94·5	5·4	96·6	7·4	1·7	6·0	*P* = 0·068	*P* = 0·085	*P* = 0·002
Central DBP (mmHg)**											
Pretest control	65·8	5·6	65·6	6·6	–	–	0·0	4·4	*P* = 0·966	–	–
Water	–	–	66·5	7·3	64·4	5·5	–2·2	5·5	*P* = 0·010	*P* = 0·006	–
Casein	–	–	67·7	5·8	65·7	6·0	–1·8	5·1	*P* = 0·048	*P* = 0·034	*P* = 0·540
Skimmed milk	–	–	66·6	6·5	66·2	5·7	–0·3	5·5	*P* = 0·705	*P* = 0·267	*P* = 0·061
Whey	–	–	66·2	5·5	67·1	5·2	0·9	5·2	*P* = 0·276	*P* = 0·875	*P* = 0·003

SBP, systolic blood pressure; –, no measurements were performed; DBP, diastolic blood pressure.

* There were no differences between the four test-drink groups at baseline (week 0).

† For test drinks, calculated as the change from week 0 to week 12; in the pretest control group, calculated as the change from week –12 to week 0.

‡ Level of significance between week 0 and week 12 for the test-drink groups and between week –12 and week 0 for the pretest control group.

§ Level of significance for difference over the intervention between the test-drink group and the pretest control group.

|| Level of significance for difference over the intervention between the milk-based test drink and water.

¶ For brachial blood pressures, at week –12 (*n* 32); at week 0 (water (*n* 50), casein (*n* 47), skimmed milk (*n* 48), whey (*n* 48)); at week 12 (water (*n* 50), casein (*n* 36), skimmed milk (*n* 44), whey (*n* 43)).

** For central blood pressures, at week –12 (*n* 30); at week 0 (water (*n* 48), casein (*n* 44), skimmed milk (*n* 46), whey (*n* 45)); at week 12 (water (*n* 47), casein (*n* 36), skimmed milk (*n* 42), whey (*n* 42)).

Central DBP decreased from week 0 to week 12 by 3·2 % within the water group (*P* = 0·010) and by 2·6 % within the casein group (*P* = 0·048). Also, there was a decrease in central DBP in the water group (*P* = 0·006) and the casein group (*P* = 0·034) compared with the pretest control group. The decreasing effects of water and casein remained significant compared with the pretest control group in the model adjusted for BMI, Tanner stage, age and sex (*P* = 0·008 and *P* = 0·017, respectively). There was an increase in central DBP in the whey group compared with the water group (*P* = 0·003) and the effect persisted in the adjusted model (*P* = 0·008). Finally, there were no significant changes in central DBP in the skimmed milk group.

There were no significant effects of skimmed milk or casein on brachial SBP or within the whey group compared with baseline. However, brachial SBP increased in the whey group compared with the water group (*P* = 0·009). Also, in the adjusted model, the increasing effect of whey on brachial SBP was significant compared with the water group (*P* = 0·012).

The whey group had a significantly increased central SBP compared with the water group (*P* = 0·002) and the effect remained significant in the model adjusted for BMI, sex, Tanner stage and age (*P* = 0·004). There were no significant differences in central SBP in the water, skimmed milk or casein groups.

BMI was a positive predictor in all models with BP as an outcome (*P* < 0·001). There were no interactions between sex and test-drink groups for any of the BP measurements.

### Vascular function and inflammation

CRP concentrations >10 mg/l were considered to be caused by acute inflammatory diseases^(^^34^^)^ and were not used in statistical analysis (five at week 0 (three water, one casein and one skimmed milk), nine at week 12 (one water, two casein, two skimmed milk and four whey)). CRP increased from week 0 to week 12 within the casein group (*P* = 0·041) (Table 5). However, there were no significant differences between any of the groups in CRP, PWV or Aix compared with the pretest control or compared with the water group.

**Table 5.** Measurements of arterial stiffness and the concentration of C-reactive protein (CRP) in the pretest control group and in those drinking water, casein, skimmed milk or the whey drink* (Mean values and standard deviations or medians and interquartile ranges (IQR))

	Week –12		Week 0		Week 12		Change†				
	Mean	sd	Mean	sd	Mean	sd	Mean	sd	Paired test‡	Treatment§	Treatment‖
PWV (m/s)¶											
Pretest control	4·65	0·58	4·66	0·65	–	–	–0·04	0·46	*P* = 0·611	–	–
Water	–	–	4·81	0·72	4·79	0·59	–0·04	0·51	*P* = 0·647	*P* = 0·787	–
Casein	–	–	4·85	0·68	4·86	0·81	0·06	0·46	*P* = 0·469	*P* = 0·746	*P* = 0·517
Skimmed milk	–	–	4·83	0·88	4·75	0·64	–0·02	0·73	*P* = 0·899	*P* = 0·855	*P* = 0·932
Whey	–	–	4·62	0·60	4·68	0·61	0·08	0·49	*P* = 0·265	*P* = 0·797	*P* = 0·544
Aix (%)**											
Pretest control	0·27	12·21	–0·92	12·08	–	–	–0·34	10·09	*P* = 0·860	–	–
Water	–	–	1·59	9·53	–0·66	10·18	–2·12	9·96	*P* = 0·152	*P* = 0·869	–
Casein	–	–	–2·23	8·16	–1·02	9·11	1·60	9·00	*P* = 0·300	*P* = 0·323	*P* = 0·333
Skimmed milk	–	–	–0·53	11·19	–3·02	11·36	–2·00	9·42	*P* = 0·182	*P* = 0·673	*P* = 0·489
Whey	–	–	–2·09	8·26	–1·65	9·47	–0·60	10·57	*P* = 0·719	*P* = 0·670	*P* = 0·753
CRP (mg/l)††											
Pretest control									*P* = 0·117	–	–
Median	0·28		0·37		–		0·05				
IQR	0·05–0·81		0·14–1·15		–		–0·15 to 0·50				
Water									*P* = 0·714	*P* = 0·315	–
Median	–		0·29		0·31		0·00				
IQR	–		0·11–0·87		0·11–1·01		−0·12 to 0·25				
Casein									*P* = 0·041	*P* = 0·885	*P* = 0·360
Median	–		0·34		0·36		0·01				
IQR	–		0·09–1·13		0·11–1·87		0·00 to 0·52				
Skimmed milk									*P* = 0·063	*P* = 0·880	*P* = 0·190
Median	–		0·21		0·51		0·04				
IQR	–		0·05–0·79		0·22–1·20		–0·19 to 0·53				
Whey									*P* = 0·209	*P* = 0·827	*P* = 0·383
Median	–		0·53		0·44		0·05				
IQR	–		0·11–1·11		0·16–1·21		–0·11 to 0·31				

PWV, pulse wave velocity; –, no measurements were performed; Aix, augmentation index.

* There were no differences between the four test-drink groups at baseline (week 0).

† For test drinks, calculated as the change from week 0 to week 12; in the pretest control group, calculated as the change from week –12 to week 0.

‡ Level of significance between week 0 and week 12 for the test-drink groups and between week –12 and week 0 for the pretest control group.

§ Level of significance for difference over the intervention between the test-drink group and the pretest control group.

|| Level of significance for difference over the intervention between the milk-based test drink and water.

¶ For PWV, at week –12 (*n* 31); at week 0 (water (*n* 48), casein (*n* 45), skimmed milk (*n* 42), whey (*n* 47)); at week 12 (water (*n* 44), casein (*n* 32), skimmed milk (*n* 36), whey (*n* 42)).

** For Aix, at week –12 (*n* 30); at week 0 (water (*n* 48), casein (*n* 44), skimmed milk (*n* 46), whey (*n* 45)); at week 12 (water (*n* 47), casein (*n* 36), skimmed milk (*n* 42), whey (*n* 42)).

†† For CRP, at week –12 (*n* 32); at week 0 (water (*n* 47), casein (*n* 46), skimmed milk (*n* 47), whey (*n* 48)); at week 12 (water (*n* 48), casein (*n* 34), skimmed milk (*n* 41), whey (*n* 39)).

## Discussion

In the present study, we have shown that high intakes of casein for 12 weeks decrease brachial and central aortic DBP in overweight adolescents compared with baseline and compared with a control group consuming no test drink. Also, the study shows that whey protein increases brachial SBP, central aortic SBP and central DBP compared with drinking water. Otherwise, the study shows no effects on inflammation or vascular function of skimmed milk, whey or casein compared with drinking water or compared with consuming no test drink in overweight but otherwise healthy adolescents.

Central aortic BP is more strongly related to vascular disease than brachial pressure^(^^17^^,^^35^^)^ and brachial SBP tend to overestimate central aortic SBP especially in young subjects because of pronounced amplification as the pulse wave moves towards the periphery^(^^35^^)^. Therefore, in the present study of adolescents we used radial artery applanation tonometry and pulse wave analysis to derive the central aortic pressures. We found that central aortic BP correlated well with brachial BP; however, central aortic SBP was lower than brachial SBP, which is consistent with other studies, and which is most pronounced in young subjects^(^^17^^,^^35^^)^. In a separate paper using data of the present study we have shown that whey and casein increased body weight compared with the pretest control group and compared with drinking water^(^^25^^)^. Thus, in the present study brachial and central aortic DBP decreased in our casein group despite an increase in body weight and the effect was stronger after adjustment for BMI. The reduction of 2·7 % in brachial DBP within the casein group is consistent with a recent study in overweight adults which also showed a 3 % reduction in brachial DBP following both whey and casein consumption^(^^15^^)^. Also, a study in pre-hypertensive adults showed reductions in both SBP and DBP following 4 weeks with a casein-derived protein hydrolysate supplement^(^^12^^)^.

We found reductions of casein in both central aortic DBP and brachial DBP compared with the pretest control; however, the findings were most significant for central aortic BP, which we speculate may be due to smaller variations within the repeated measures. On the other hand, casein may also have a more pronounced effect on central aortic BP. This is supported by studies showing that blood pressure-lowering drugs can have the same effect on brachial BP but different effects on central aortic pressures^(^^36^^)^. The present results are backed up by a recent study in hypertensive adults, which showed that casein hydrolysate tablets containing the milk-derived tripeptides Val-Pro-Pro (valine-proline-proline) and Ile-Pro-Pro (isoleucine-proline-proline) improve central aortic BP^(^^18^^)^.

Each component of BP seems to be associated with CVD risk^(^^37^^)^; however, DBP seems to be the strongest predictor of CVD in young individuals below 50 years^(^^38^^)^. Therefore, the reductions of 1·8 mmHg in DBP following casein consumption seen in the present study may probably be beneficial for younger subjects in terms of reducing CVD risk later in life.

The mechanism behind the improvements in DBP following casein consumption may occur through inhibition of the ACE system. ACE is a multifunctional enzyme that plays a key role in the regulation of BP. The enzyme system catalyses the conversion of angiotensin I to angiotensin II, which is a potent vasoconstrictor and it degrades bradykinin, which is a potent vasodilator. *In vitro* studies have found ACE-inhibitory peptides in the amino acid sequence of both whey-derived proteins and casein^(^^19^^)^.

We were surprised to see an increased effect of whey on brachial SBP, central DBP and central SBP compared with water since studies in adults have shown no effects of whey^(^^39^^)^ or reduced BP following whey consumption^(^^13^^–^^15^^)^. The effects of whey on BP seen in the present study were only slightly attenuated but remained significant after adjustment for BMI, indicating that the increased body weight may only partly explain the increased BP. There may be several explanations for the inconsistency between the studies. First, we only found significantly increased effects of whey on BP when compared with the water group, which showed reductions in all BP measurements, though only significant for central DBP. In comparison, other studies did not include a control group that did not consume a whey product^(^^13^^)^ or they used a glucose control group^(^^15^^)^, which may adversely affect CVD risk factors. Second, the majority of our adolescents were normotensive, considering BP levels of ≥120/80 mmHg as pre-hypertensive^(^^40^^)^, and it may be that whey only reduces BP in hypertensive subjects. Thus, the study by Fluegel *et al.*^(^^13^^)^ divided the study group according to BP levels and did not find an effect of whey on BP in normotensive but only in the pre- and hypertensive subjects and most of the studies showing reduced effects were conducted in pre- or hypertensive subjects^(^^13^^,^^14^^)^. Finally, an explanation for the discrepancy may be that the effect of whey on BP differs during the life span and to our knowledge no other studies have been conducted in adolescents.

In a previous cross-sectional study using the present baseline data, we found a trend toward an inverse association between milk intake and arterial stiffness measured by PWV^(^^24^^)^. Therefore, we hypothesised that increasing the intake of skimmed milk would have a beneficial effect on PWV and Aix as measures of arterial stiffness. The hypothesis was supported by studies showing reductions in Aix following consumption of whey or peptide milk for 12 weeks^(^^15^^,^^41^^)^. However, the present study does not add support to the hypothesis that increasing milk intake improves arterial stiffness. Also, we did not find any effects of skimmed milk, whey or casein on CRP compared with the pretest control group or compared with drinking water, which is consistent with a recent study using whey and casein in overweight adults^(^^15^^)^ nor did we find any effects on blood lipids.

### Strength and limitations

The strength of the study is the double-blinded (on the milk-based test drinks) randomised design conducted in a large sample of overweight adolescents. The study was powered to detect a relatively small difference of 0·4 sd in any outcome variable should it exist and dietary interventions examining the effects on PWV, Aix and central BP are rarely reported in the paediatric literature. Water may not be an appropriate control since studies have shown improvements in weight loss^(^^26^^)^ and the present data also show that all the BP measures were reduced within the water group, though only central DBP was significantly reduced. Other studies have used carbohydrate-rich drinks^(^^15^^,^^20^^,^^42^^)^. However, these may in themselves adversely affect the metabolic syndrome risk factors. Therefore we decided also to use a pretest control group which strengthens the conclusions of the present study because the data show no changes in any of the outcomes within the pretest control group, indicating that this may be an appropriate control group.

A limitation is that we had difficulties in recruiting the overweight adolescents with low habitual milk intakes. We aimed for fifty adolescents in each of the test-drink groups, and, of those, fifty adolescents should be followed for 3 months before starting the intervention as the pretest control group. We recruited for the pretest control group in the autumn of 2009 using the birth years 1995 and 1996, but only thirty-two adolescents were enrolled and measured at week –12. Due to the difficulties in obtaining enough participants, we only recruited for the test-drink groups in 2010 and we used the birth years 1997 and 1998 because the older adolescents had been invited previously. Therefore, the pretest control group ended up being approximately 6 months older when starting the study (week –12) compared with the mean age of the four test-drink groups. We performed analyses with adjustments for age, Tanner stage and sex for all the intervention outcome variables, which did not change the results remarkably. Also, it is a limitation that the pretest control group was only recruited in the autumn. Thus, seasonal variations in the outcomes or in lifestyle may potentially have biased the comparisons between the test-drink groups and the pretest control group. However, neither BP nor blood lipids changed within the 12-week period in the pretest control group. In addition, the smaller number of subjects in the pretest control group may have reduced the probability of detecting differences between this and the other groups due to lower power. Furthermore, a limitation of the present study focusing on the effects of milk-based test drinks on haemodynamic variables is that skimmed milk, whey and casein previously were found to increase body weight. We hypothesised that increasing the intake of skimmed milk, whey and casein would reduce body weight in overweight adolescents with habitual low milk intakes. Thereby we assumed that the children were able to regulate their energy intake as seen in other *ad libitum* studies with dairy products in children^(^^43^^–^^46^^)^. We also assumed that the increased protein intake would increase satiety and hence decrease energy intake as seen in adults^(^^47^^–^^49^^)^. The hypothesis was supported by an adult study showing that a high-protein diet in *ad libitum* settings increases weight loss^(^^50^^)^. However, we performed the analyses with adjustments for BMI, which did not change the results remarkably. Finally, it was not possible to obtain the same mineral content in the test drinks, which potentially may have affected the results. Thus, Ca has been found to enhance vasorelaxation^(^^51^^)^ and to improve the blood lipid profile^(^^52^^,^^53^^)^. Also Na seems to adversely affect BP and arterial function whereas K and Mg may have beneficial effects on vascular function and BP^(^^54^^,^^55^^)^. In our test drinks, Mg concentrations were slightly different between the test drinks. K content was highest in skimmed milk whereas Na content was highest in the casein drink. Thus, it seems unlikely that the differences in Na and K concentrations could explain the different BP effects of the test drinks.

In conclusion, the present study demonstrated that a high intake of casein improves brachial and central aortic DBP in overweight adolescents despite casein in our previous study having been shown to increase body weight. Thus, the reducing effects on DBP seem to be independent of body size, and since raised DBP in subjects aged 20–50 years is known to increase the risk of CVD^(^^38^^)^, casein may be a beneficial non-pharmacological component for younger overweight subjects in terms of reducing the long-term risk of CVD. We also showed that whey protein increases BP compared with drinking water; however, water may not be an optimal control group since BP was reduced within this group and therefore more interventions in children are needed to clarify the effects of whey protein.
